# Identification of Cardiac MRI and Bio-Marker Thresholds for One-Year Survival in Pre-Capillary Pulmonary Hypertension: Prospective Study

**DOI:** 10.3390/medicina56040167

**Published:** 2020-04-09

**Authors:** Lina Padervinskiene, Deimante Hoppenot, Ausra Krivickiene, Birute Gumauskiene, Irena Nedzelskiene, Paulius Simkus, Skaidrius Miliauskas, Antanas Jankauskas, Algidas Basevicius, Egle Ereminiene

**Affiliations:** 1 Department of Radiology, Medical Academy, Lithuanian University of Health Sciences, LT 44307 Kaunas, Lithuania; pauliusimkus@gmail.com (P.S.); antanas.jankauskas@lsmuni.lt (A.J.); algidas.basevicius@lsmuni.lt (A.B.); 2 Department of Pulmonology, Medical Academy, Lithuanian University of Health Sciences, LT 44307 Kaunas, Lithuania; deimante.hoppenot@lsmuni.lt; 3 Department of Cardiology, Medical Academy, Lithuanian University of Health Sciences, LT 44307 Kaunas, Lithuania; ausra.krivickiene@lsmuni.lt (A.K.); birute.gumauskiene@lsmuni.lt (B.G.); skaidrius.miliauskas@lsmuni.lt (S.M.); egle.ereminiene@lsmuni.lt (E.E.); 4 Department of Dental and Oral Diseases, Medical Academy, Lithuanian University of Health Sciences, LT 44307 Kaunas, Lithuania; irena.nedzelskiene@lsmuni.lt

**Keywords:** pulmonary hypertension, cardiac magnetic resonance, right ventricle, feature tracking

## Abstract

*Background and objectives*: Non-invasive imaging of the heart has an important place in the diagnosis and management of pulmonary arterial hypertension (PAH). The aim of this study was to establish the thresholds of cardiac magnetic resonance imaging (CMRI)-derived biventricular deformation, function parameters, and levels of N-terminal pro brain natriuretic peptide (NT-proBNP) for the prediction of survival of pre-capillary pulmonary hypertension (PHprecap) patients. *Materials and Methods*: In total, 64 incident PHprecap cases, who underwent CMRI, were consecutively enrolled in a prospective cohort study. Patients underwent a systemic evaluation, including measurement of NT-proBNP, two-dimensional (2D) echocardiography, six-minute walk test (6MWT), CMRI with feature tracking (FT), and right-heart catheterization (RHC). Patients were divided into two groups according to one-year survival (survival and non-survival groups). Survival analysis was performed. *Results*: One-year survival was 79.6%. The distribution between age, sex, mean pulmonary artery pressure (mPAP), New York Heart Association (NYHA) functional class, and 6MWT did not differ between the groups. Survival was significantly lower in the PAH group associated with connective tissue disease (CTD-PAH), where 44% (*n* = 4) of patients died during the first year. Univariate analysis revealed that severely reduced right-ventricle (RV) ejection fraction (EF) <25.5%, left-ventricle global longitudinal strain (LV GLS) >−14.18%, and right pulmonary artery (RPA) relative area change (RAC) <19%, and severely increased NT-proBNP level >1738 (ng/L) indicate an increased risk of death in PH_precap_ patients. *Conclusions*: Impaired RV systolic function and LV global longitudinal strain, decrease of pulmonary artery distensibility, and CTD-PAH etiology, together with high NT-proBNP level, impair prognosis in pre-capillary PH patients. These findings are important for the risk stratification and management of pre-capillary pulmonary hypertension patients.

## 1. Introduction

Pulmonary hypertension (PH) is a progressive disease affecting pulmonary arteries that is associated with right-ventricular (RV) failure and bad prognosis without appropriate treatment [1]. However, mortality remains high despite specific pulmonary arterial hypertension (PAH) treatment [2]. PAH pathophysiology and the course of the disease are complicated, and there are still many unanswered questions. Therefore, the search of predictors for PAH outcomes remains essential [3]. Non-invasive imaging of the heart has an important place in the diagnosis and management of PAH [1,4,5,6,7,8]. PAH may be suspected, guiding clinical course and echocardiographic findings, when elevated systolic RV pressure and right heart chamber dilatation are determined [9]. Cardiac magnetic resonance imaging (CMRI) is a gold standard for RV volumetric and functional evaluation [10]. As right heart failure is critically important in PAH outcomes, precise measurements need to be performed at the time of diagnosis and during follow-up [11].

To the best of our knowledge, the significance of CMRI-derived RV volumetric and functional parameters, together with feature tracking evaluation of biventricular deformation indices, pulmonary arterial distensibility, and specific biomarker values in pre-capillary PH during one-year follow-up, were not previously investigated. The aim of this study was to establish the thresholds of CMRI-derived biventricular deformation, function parameters, and levels of N-terminal pro brain natriuretic peptide (NT-proBNP) for the prediction of survival of pre-capillary pulmonary hypertension (PH_precap_) patients.

## 2. Materials and Methods

Between November 2012 and October 2019, all 64 incident PH_precap_ cases, who underwent CMRI, were consecutively enrolled in a prospective cohort study. PH_precap_ was confirmed by right heart catheterization (mean pulmonary artery pressure (mPAP) 61.22 ± 18.5 mmHg, pulmonary arterial wedge pressure ≤15 mmHg). Fifteen patients in total were excluded: six for co-morbidities that could affect survival, such as underlying cardiomyopathy and valvular heart disease; three for shortness of breath during CMRI examination; four for low-quality CMRI studies due to arrhythmia; two for claustrophobia. Finally, 49 patients were included in the study. The characteristics of the patients at the time of their initial visit were collected from the medical records. After the diagnosis of PH_precap_, all patients underwent treatment with the specific therapy available in our country for PAH. All patients with chronic thromboembolic PH (CTEPH) were non-operable and received conservative treatment (riociguat/sildenafil). The study protocol was approved by the Regional Biomedical Research Ethics Committee (identifier (ID) No. BE-2-23). The study was registered in Protocol Registration and Results System (ClinicalTrials.gov ID no. NCT03377673). Informed consent was obtained from each patient. Patients underwent systemic evaluation, including measurement of NT-proBNP, two-dimensional (2D) echocardiography, six-minute walk test (6MWT), cardiac MRI with feature tracking, and right heart catheterization. The datasets used and analyzed during the current study are available from the corresponding author on reasonable request.

### 2.1. Cardiac MRI Measurements

#### Cardiac MRI Volumetric and Functional Measurements

**Figure 1:**
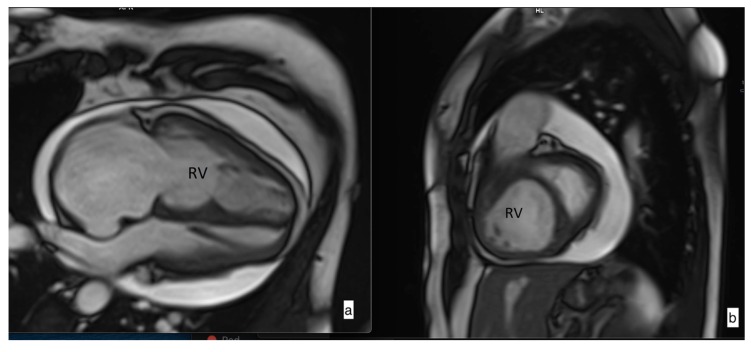
Steady-state free precession (SSFP) cine imaging by cardiac magnetic resonance imaging of a patient with pulmonary arterial hypertension. SSFP in the four-chamber (**a**) and short-axis (**b**) orientation at early diastole showing right heart chamber dilatation and leftward ventricular septal bowing due to increased pressure in the right ventricle (RV). Pericardial effusion is also noticed.

Cardiac MRI was performed using a 1.5-T whole-body system (Siemens Aera, Siemens Medical Solutions; Erlangen, Germany). Image analysis was performed on an MR analysis software system (syngo.via; Siemens Healthcare). Four-chamber (4Ch) and short-axis (SA) cine images (Figure 1) were acquired using a retrospectively cardiac gated multi-slice steady-state free precession (SSFP) sequence. Right and left endocardial and epicardial surfaces were manually traced from the stack of axial images in the SA plane, which were acquired with a slice thickness of 8 mm and a 2 mm inter-slice gap or 10 mm with no inter-slice gap, covering both ventricles from base to apex. The end-diastolic and end-systolic volumes of both ventricles were obtained. From these volumes, ventricle stroke volume (SV) and ejection fraction (EF) were calculated. The end-diastolic, end-systolic, and stroke volumes of both ventricles were indexed for body surface area. For calculation of RV mass, the interventricular septum was considered as part of the left ventricle (LV), and all RV papillary muscles were included in mass analysis. Right-atrial (RA) and left-atrial (LA) area were measured by manual tracing of endocardial maximal contour in the 4Ch image at ventricular end-systole.

### 2.2. Pulmonary Artery Distensibility

**Figure 2:**
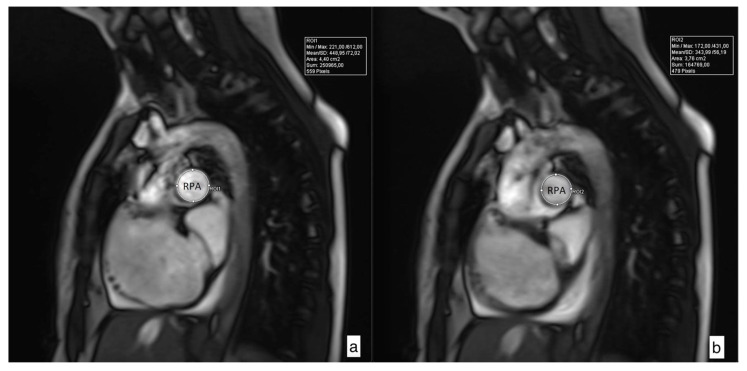
Cross-sectional images of maximal (**a**) and minimal (**b**) right pulmonary artery (RPA) area used for determination of relative area change (RAC) according to the following formula: RAC = ((maximum area − minimum area)/minimum area) × 100%. RPA RAC in this case is 17.02%.

Pulmonary artery distensibility is defined as the relative change in the cross-sectional artery area throughout the cardiac cycle multiplied by the pulse pressure required to induce that change. Since the main pulmonary artery pulse pressure is not readily known, the pulsatility or relative area change (RAC) is used as a surrogate marker of MPA stiffness [12]. Maximal and minimal areas of pulmonary arteries were measured, and relative area change (RAC) was defined by the following equation: RAC = ((maximum area − minimum area)/minimum area) × 100% (Figure 2) [13]. These measurements were performed for the main pulmonary artery (MPA), right pulmonary artery (RPA), and left pulmonary artery (LPA).

### 2.3. Late Gadolinium Enhancement (LGE) Assessment

Delayed-enhancement images for detection of hyperenhancement were obtained ~10 min after injection of double-dose intravenous gadobutrol (0.2 mmol/kg) using a segmented inversion-recovery prepared fast gradient echo sequence. Non-ischemic LGE pattern was considered to be present if the signal intensity in the myocardium at the ventricle insertion points (VIP) or extended to the intraventricular septal was greater than or equal to that seen in the blood pool, present in two consecutive slices, and clearly present within the myocardium when compared against a matching SSFP cine image.

### 2.4. Feature Tracking Mechanical Analysis

**Figure 3:**
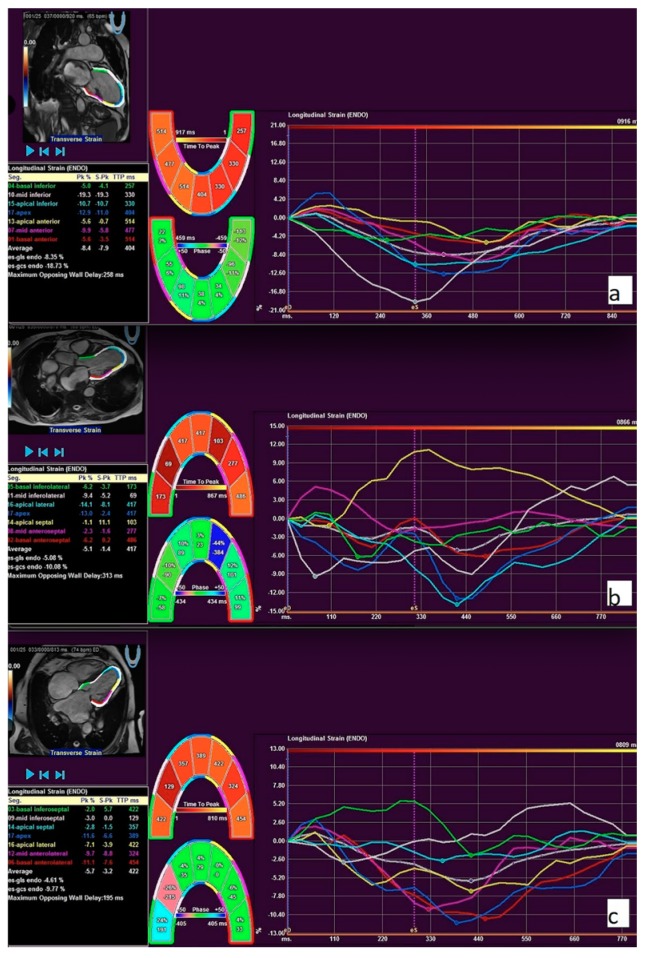
Feature tracking (FT) strain curves of two-chamber (**a**), three-chamber (**b**), and four-chamber (**c**) long-axis views. The global longitudinal strain was calculated by averaging all long-axis strain parameters. The example of longitudinal strain measurements in a patient with pulmonary hypertension.

CMR images were analyzed using a commercial feature tracking (FT) software package (Medis Suite QStrain 2.0; Medis Medical Imaging Systems bv, Leiden, the Netherlands). Two-, three-, and four-chamber and short-axis cine images were uploaded into the software and were used for the analysis of both ventricle longitudinal and LV circumferential strain and strain rate analysis. FT analysis was done semi-automatically by delineating the endocardial surfaces throughout the cardiac cycle. The contours were checked and manually adjusted if needed. LV global longitudinal strain (GLS) and strain rate (GLSR) were calculated by averaging the strain curves of two-chamber, three-chamber, and four-chamber long-axis views (Figure 3). LV global circumferential strain (GCS) and strain rate (GCSR) were calculated by averaging the strain curves of basal, mid, and apical segments obtained from the short-axis views. RV GLS and GLSR were calculated in the cardiac four-chamber long-axis view.

### 2.5. Data Analysis

Statistical analysis was performed using SPSS 22.0 package (SPSS, Chicago, IL, USA). Continuous variables were summarized by mean ± SD or median (interquartile range), as appropriate. For continuous variables, differences between the two groups were compared using the non-parametric Mann-Whitney U test. A *p*-value <0.05 was considered significant. A chi-square (χ^2^) test was used for qualitative data. Spearman’s rank correlation coefficient was used to summarize the strength and direction of a relationship between two variables. In order to assess minimally false negative and minimally false positive results with greatest accuracy, the method of the ROC (receiver operating characteristics) curve was used. There were two steps to the survival analysis: univariable and binary logistic regression analysis. Variables identified in the univariable analyses were entered into a binary logistic regression model. Proportional hazards assumptions were confirmed with a Kolmogorov-type supreme test. Hazard ratio (HR) and the difference between matched groups were expressed with 95% confidence interval (95% CI). For CMR, volumetric measurements indexed for body surface area were corrected for age and sex.

## 3. Results

### Patient Characteristics

Clinical and demographic characteristics are shown in Table 1. In total, 49 patients with PH_precap_ fulfilled the criteria. Patients were divided into two groups according to one-year survival (survival/non-survival). The observed one-year survival from the date of enrolment was 79.6%; all patients died due to cardiopulmonary events. The distribution between age, sex, mean PA pressure, New York Heart Association (NYHA) class, and 6MWT did not differ between groups. The majority of patients (35/49 patients; 71.4%) were in group 1 of the European Society of Cardiology (ESC)/European Respiratory Society (ERS) Guidelines classification of PH, with the remaining patients having chronic thromboembolic pulmonary hypertension (group 4, *n* = 14). All patients from group 1 underwent specific treatment (i.e., phosphodiesterase type 5 inhibitors, endothelin antagonist, prostacyclin analogues, 64% monotherapy, 36% combination therapy). All CTEPH patients included into analysis were non-operable and received medical treatment. Survival was significantly lower in the PAH group associated with connective tissue disease (patients with systemic sclerosis) (CTD-PAH), where 44% (*n* = 4) of patients died during the first year. In the CTD-PAH group, the odds ratio to die was found to be 5.833 (1.138–29.899). Furthermore, NT-proBNP level was significantly higher in the non-survival group (*p* = 0.016) (Table 1). NT-proBNP level correlated with a poor PAH outcome variable, i.e., RV ejection fraction (EF) (*r* = 0.349, *p* = 0.016). Non-ischemic LGE pattern was evaluated in 43 patients. LGE was found in 79.1% of patients (*n* = 34) and did not differ between the groups (80.6% (*n* = 29) vs. 71.4% (*n* = 5), *p* = 0.274).

Analysis of CMR volumetric and functional parameters demonstrated that baseline RV EF was significantly lower in the one-year non-survival group (30.5% (21.5–40.75) vs. 38.0% (31.0–46.0), *p* = 0.039, respectively) (Table 2). Areas of both atria, indices of LV and RV volumes, and LV EF did not differ between groups. LV global longitudinal strain (GLS) was significantly reduced in the non-survival group (−12.17% (−18.57–(−6.85) vs. −17.61% (−19.78–(−15.11)), *p* = 0.021, respectively) (Table 2). LV GLS correlated with RV EF (*r* = −0.351, *p* = 0.029). Other biventricular mechanical parameters were lower in the non-survival group but did not meet statistical significance.

Right pulmonary artery RAC was significantly lower in the non-survival group (11.89% (0.0–15.77) vs. 19.36% (11.48–23.79), *p* = 0.046) (Table 3). Other baseline pulmonary arteries distensibility values did not differ between the groups.

Univariate analysis (Table 4) revealed that severely reduced RV EF <25.5% and LV GLS >−14.18%, as well as severely increased NT-proBNP level >1738 (ng/L), indicated an increased risk of death.

Two models were developed to predict death in one year: (1) LV global longitudinal strain; (2) RV ejection fraction in the presence of CTD-PAH (Table 5).

## 4. Discussion

Several studies were conducted to understand mortality rates in PAH which demonstrated that one-year survival in PAH patients ranged from 68% to 91% [14,15,16,17,18]. Our cohort’s one-year survival was 79.6%. These differences might be due to different years of research and specific treatment availability, as well as due to various clinical conditions at the time of inclusion in the study. In our cohort, most of the patients were with advanced PH_precap_ disease (NYHA class 3 and 4). Moreover, pulmonary hypertension associated with connective tissue disease (CTD-PAH) was associated with the worst one-year survival rate, as compared with other etiology of pre-capillary PH, and these data agree with other studies.

Main prognostic parameters in PAH are based on clinical symptoms (clinical signs of RV failure, progression of symptoms, syncope), World Health Organization (WHO) functional class, 6MWT, laboratory (NT-proBNP level), hemodynamics, and cardiopulmonary exercise testing, as well as different imaging modalities such as echocardiography or CMR [9]. However, data on non-invasive imaging parameters associated with short-term or long-term prognosis in PAH patients are scarce. Compared to other studies investigating this subject, our study adds to the understanding of the prognostic significance of CMR basic and additional novel functional biventricular parameters, based on feature tracking modality. In this study, we analyzed indices of the right- and left-ventricular geometry, function, and deformation, together with pulmonary artery distensibility.

RV dysfunction is related to the severity of PAH and clinical status of the patient [19]. Lewis and colleagues recently evaluated and validated right-ventricular CMR parameters for risk stratification in PAH [20]. In this study, patients were divided into low, intermediate, and high risk according one-year mortality, and RV EF was as follows: >54%, 37%–54%, and <37%. Based on 2015 ESR/ERS recommendations of PAH risk assessment [9], our subjects met intermediate or high-risk criteria. Our data showed that median RV EF was 30.5% in non-survival group patients. However, the threshold of RV EF below 25.5% was associated with greater than eight-fold risk of death risk in our study patients. RV systolic function is an important prognostic value in PH, and this was proven by several studies. One decade ago, van de Veerdonk et al. performed an elegant study where they already showed that an RV EF cut-off <35% and a decrease in RV EF during PAH patient follow-up were related to higher mortality risk [1]. Nevertheless, other studies did not find the prognostic significance of RV EF, but they found RV EDVI and RV ESVI [6] or RV ESVI alone [21] as predictors of mortality. The latter studies showed the adverse prognostic impact of RV volumetric measurements in patients with PAH. However, RV end-diastolic and systolic volumes did not differ between the groups (*p* = 0.3 and *p* = 0.07, respectively) in our study, possibly because of the small sample size and the heterogeneous population enrolled in the study.

CMR allows non-invasive assessment of pulmonary arterial distensibility [9]. Several studies highlighted the prognostic value of pulmonary arterial distensibility in the prognosis of PAH [22]. Paz et al., two decades ago, published normal values of distensibility of pulmonary arteries (MPA 25.6% ± 10.7%, RPA 21.4% ± 10.7%, and LPA 24.5% ± 7.8%) [23]. Our data show clearly that our patients presented severely reduced RAC of all pulmonary arteries. Gan and colleagues also found that RPA RAC was significantly lower in the non-survival group, and patients with RAC ≤16% had a significantly lower survival rate than those with RAC >16% [24]. Our data agree with Gan et al.’s findings, as we revealed a significant difference in RPA RAC between survival and non-survival groups (*p* = 0.046), and a cut-off of 19.075% was found to be a predictor of mortality in our patient cohort. This could be related to the fact that correct measurement of the main pulmonary artery cross-sectional area is quite difficult due to its conical shape and cardiac motion in the through-plane direction, which may result in false dilatation or contraction, and this could be the reason for the inability to find significant differences. Unlike MPA, RPA is a relatively straight tube and it is mostly affected by cardiac motion in the transversal direction, which does not result in artificial distension or constriction [24]. Thus, measurements of RPA area are more reliable when compared with left and main PA measurements.

The importance of left-ventricular parameters in patients with pulmonary hypertension was shown in numerous papers [6,19,21,25,26]. However, still there is no unequivocal opinion about CMRI parameters which could help to stratify the risk in pre-capillary pulmonary hypertension patients. Van Wolferen et al. concluded that low stroke volume and impaired left-ventricular filling independently predict mortality [19]. Yamada et al. suggested that LV mass index, as well as end-diastolic and end-systolic volume indices of both ventricles, predicted the risk for hospitalization [6]. Swift and colleagues found that corrected LV SV predicted an adverse outcome in idiopathic PAH patients [21]. In the current study, LV SVI also had a tendency to be lower in the non-survival group (31.5 (25.5–47.5) vs. 36.0 (31.0–45.0) mL/m^2^, *p* = 0.275, respectively), but did not reach statistical significance. Ventricular interaction mediated by the interventricular septum impairs the LV’s possibility to expand, contributing to a decreased LV global longitudinal strain. In a previous study, we found a correlation between LV systolic functions and LV global longitudinal strain with RV dysfunction and poor clinical outcomes in long-term follow-up [25]. Kallianos et al. compared PH patients’ LV strain parameters with controls and found that LC GCS was significantly different [26]. We indicated the prognostic significance of LV GLS >−14.18% in the overall PH_precap_ cohort. The present study highlights that, even in short-term follow-up, mechanical estimates of LV strains may predict an increased risk of death in patients with PH_precap_.

Our data show that increased NT-proBNP could also be one of mortality predictors in PH. We found that patients with NT-proBNP level >1738 (ng/L) had reduced survival over one-year follow-up. Thus, our findings are in agreement with the COMPERA registry, where patients with NT-proBNP level >1400 (ng/L) meet the criteria of the high-risk group [2].

## 5. Conclusions

Impaired RV systolic function, LV global longitudinal strain, decreased pulmonary artery distensibility, CTD-PAH etiology, and a high level of NT-proBNP are associated with poor prognosis in pre-capillary PH patients. These findings are important for risk stratification and management of pre-capillary pulmonary hypertension patients.

The main limitation of this study was the small sample size, reflecting the uncommon occurrence of PAH. Sample heterogeneity was limited by only including patients with pre-capillary PH; however, underlying conditions still varied.

## Figures and Tables

**Table 1:** Clinical and demographic characteristics of all pre-capillary pulmonary hypertension (PH_precap_) patients and between groups.

Characteristics	All Patients (*N* = 49)	Survival (*N* = 39)	Non-Survival (*N* = 10)	*p*-Value
Age (years)	58.5 (46.38–70.04)	58.53 (47.67–70.64)	55.15 (45.18–68.1)	0.884
Women/men	30 (61.2%)/19 (38.8)	25 (83.3%)/14 (73.7%)	5 (16.7%)/5 (26.3%)	0.419
IPAH (*n* (%))	17 (34.7%)	14 (35.9%)	3 (33.0%)	0.363
CTD-PAH (*n* (%))	8 (16.3%)	4 (10.3%)	4 (44.0%)	0.012
Eisenmenger syndrome (*n* (%))	6 (12.8%)	5 (12.8%)	1 (10.0%)	0.404
CTEPH (*n* (%))	14 (28.6%)	13 (33.3%)	1 (10.0%)	0.073
PAH other cause (*n* (%))	4 (8.2%)	3 (7.7%)	1 (10.0%)	0.406
NYHA class 2/3/4 (*n* (%))	11 (22.44)/28 (57.12)/10 (20.4)	9 (23.04)/25 (64.0)/5 (12.8)	2 (20.0)/3 (30.0)/5 (50.0)	0.708 *
mPAP (mmHg)	61.22 ± 18.5	61.49 ± 19.1	58.75 ± 13.79	0.783
6MWT (m)	285.0 (217.5–408.0)	280.0 (240.0–405.0)	225(170.0–419.0)	0.69
NT-pro BNP (ng/L)	1738.0 (469.0–4100.0)	1472 (398.75–3322.0)	3683.0 (1902.5–6957.0)	0.016

Definition of abbreviations: IPAH—idiopathic pulmonary arterial hypertension; CTD-PAH—pulmonary hypertension associated with systemic sclerosis; CTEPH—chronic thromboembolic pulmonary hypertension; NYHA—New York Heart Association functional class; mPAP—mean pulmonary artery pressure; 6MWT—six-minute walk test; NT-pro BNP—brain natriuretic peptide. The *p*-values were determined using the Mann–Whitney U test or chi-square test *. Values are medians (interquartile range) or *n* (%). * NYHA *p* = 0.708, degrees of freedom (df) = 2.

**Table 2:** Baseline cardiac magnetic resonance (CMR) volumetric, functional, and strain (FT) parameters in all PH_pre-cap_ patients and between groups.

Parameters	All PH Patients (*N* = 49)	Survival (*N* = 39)	Non-Survival (*N* = 10)	*p*-Value
RV EDVI (mL/m^2^)	85.0 (69.0–104.5)	84.0 (68.0–101.0)	87.0 (78.0–125.75)	0.312
RV ESVI (mL/m^2^)	53.0 (43.5–70.0)	50.0 (41.0–65.0)	67.0 (47.25–82.5)	0.073
RV mass (g/m^2^)	53.0 (41.5–59.0)	53.0 (40.5–57.5)	54.0 (43.0–77.75)	0.116
RV EF (%)	37.0 (30.0–45.0)	38.0 (31.0–46.0)	30.5 (21.5–40.75)	0.039
RV GLS (%)	−13.8 (−16.6–(−10.5))	−14.15 (−16.68–(−11.53))	−10.5 (−18.0–(−8.3))	0.134
LV EDVI (mL/m^2^)	64.0 (54.0–80.5)	64.0 (55.0–81.0)	64.0 (46.5–79.0)	0.604
LV ESVI (mL/m^2^)	32.0 (20.5–38.5)	32.0 (20.0–38.0)	32.0 (22.0–41.75)	0.213
LV EF (%)	54.0 (46.0–63.0)	55.0 (46.0–63.0)	49.0 (42.25–53.75)	0.140
LV GLS (%)	−16.43 (−19.5–(−12.63))	−17.61 (−19.78–(−15.11))	−12.17 (−18.57–(−6.85))	0.021
LV GCS (%)	−31.1 (−34.7–(−25.97))	−31.6 (−35.82–(−26.39))	−29.9 (−32.28–(−21.88))	0.224
RA (cm^2^)	30.0 (24.25–35.5)	29.0 (24.0–34.0)	33.0 (26.0–39.5)	0.355
LA (cm^2^)	21.0 (17.0–27.5)	22.0 (17.0–28.0)	18.0 (15.75–22.0)	0.2

Definition of abbreviations: RV—right ventricular; EDVI—end-diastolic volume index; ESVI—end-systolic volume index; EF—ejection fraction; LV—left ventricular; GLS—global longitudinal strain; GCS—global circumferential strain RA—right atrium; LA—left atrium. The *p*-values were determined using the Mann–Whitney test. Values are medians (interquartile range).

**Table 3:** Pulmonary artery CMR distensibility parameters in all PH_precap_ patients and between groups.

Parameters	All Patients (*N* = 49)	Survival (*N* = 39)	Non-Survival (*N* = 10)	*p*-Value
MPA RAC (%)	12.88 (8.3–20.63)	13.25 (8.53–19.8)	9.26 (5.36–22.26)	0.254
RPA RAC (%)	15.81 (11.15–23.3)	19.36 (11.48–23.79)	11.89 (0.0–15.77)	0.046
LPA RAC (%)	12.32 (7.55–15.93)	12.86 (8.57–17.34)	9.45 (0.0–15.67)	0.277

Definition of abbreviations: MPA—main pulmonary artery; RPA—right pulmonary artery; LPA—left pulmonary artery; RAC—relative area change. The *p*-values were determined using the Mann–Whitney test. Values are medians (interquartile range).

**Table 4:** The relationship between one-year survival and parameter threshold values: results of the univariate analysis.

Parameter (Its Threshold Value)	Area Under the Curve (%)	Sensitivity/Specificity (%)	1-Year Survival/ Non-Survival *n* (%)	*p*-Value	Non-Survival Group OR (95% PI)
RV EF <25.5%	69.1	50.0/89.7	4 (10.3)/5 (50.0)	0.011	8.75 (1.741–43.973)
NT-proBNP >1738 (ng/L)	76.1	88.9/60.5	15 (39.5)/8 (88.9)	0.008	12.267 (1.389–108.325)
RPA RAC <19.075%	77.8	100.0/52.8	17 (47.2)/5 (100.0)	0.027	-
LV GLS >−14.183%	75.6	77.8/86.7	4 (13.3)/7 (77.8)	0.001	22.75 (3.432–150.811)

Definition of abbreviations: ROC—receiver operating characteristics; RV EF—right-ventricular ejection fraction; NT-pro-BNP—brain natriuretic peptide; RPA RAC—right pulmonary artery relative area change; LV GLS—left-ventricular global longitudinal strain. The *p*-values were determined using the Mann–Whitney test. OR—odds ratio; CI—confidence interval.

**Table 5:** Risk of death based on binary logistic regression analysis.

Regressors	OR (95% CI); *p*-Value
Model No. 1 (correct prediction 83.7%, Nagelkerke determination coefficient 0.552)	
LV GLS >−14.183	32.184 (3.145–329.341); 0.003
CTD-PAH	12.499 (1.0–165.877); 0.05
Model constant	−3.37, *p* < 0.001
Model No. 2 (correct prediction 83.7%, Nagelkerke determination coefficient 0.325)	
RV EF <25.5	10.12 (1.731–59.15); 0.01
CTD-PAH	7.033 (1.098–45.048); 0.04
Model constant	−2.442, *p* < 0.001

*Definition of abbreviations:* LV GLS—left-ventricular global longitudinal strain; CTD-PAH—pulmonary hypertension associated with connective tissue disease; RV EF—right-ventricular ejection fraction; OR—odds ratio. The *p*-values were determined using the Mann–Whitney test.

## Abbreviations

PH: pulmonary hypertension; PAH: pulmonary arterial hypertension; IPAH: idiopathic pulmonary arterial hypertension; CTD-PAH: pulmonary hypertension associated with systemic sclerosis; CTEPH: chronic thromboembolic pulmonary hypertension; PH_precap_: pre-capillary pulmonary hypertension; CMRI: cardiac magnetic resonance imaging; NT-proBNP: N-terminal pro brain natriuretic peptide; 6MWT: six-minute walking test; FT: feature tracking; RHC: right heart catheterization; mPAP: mean pulmonary artery pressure; NYHA: New York Heart Association; ESC: European Society of Cardiology; ERS: European Respiratory Society; RV: right ventricular; LV: left ventricular; RA: right atrial; LA: left atrial; EF: ejection fraction; EDV: end-diastolic volume; EDV: end-diastolic volume index; ESV: end-systolic volume; ESVI: end-systolic volume index; SV: stroke volume; SVI: stroke volume index; GLS: global longitudinal strain; GLSR: global longitudinal strain rate; GCS: global circumferential strain; GCSR: global circumferential strain rate; 4Ch: four-chamber; SA: short axis; SSFP: steady-state free precession; MPA: main pulmonary artery; RPA: right pulmonary artery; LPA: left pulmonary artery; RAC: relative area change; LGE: late gadolinium enhancement; VIP: ventricle insertion points; HR: hazard ratio; CI: confidence interval.

## References

[1] Van De Veerdonk M.C., Kind T., Marcus J.T., Mauritz G.J., Heymans M.W., Bogaard H.J. (2011). Progressive right ventricular dysfunction in patients with pulmonary arterial hypertension responding to therapy. J. Am. Coll. Cardiol..

[2] Hoeper M.M., Kramer T., Pan Z., Eichstaedt C.A., Spiesshoefer J., Benjamin N. (2017). Mortality in pulmonary arterial hypertension: Prediction by the 2015 European pulmonary hypertension guidelines risk stratification model. Eur. Respir. J..

[3] Stacher E., Graham B.B., Hunt J.M., Gandjeva A., Groshong S.D., McLaughlin V.V., Jessup M., Grizzle W.E., Aldred M.A., Cool C.D.T.R. (2012). Modern age pathology of pulmonary arterial hypertension. Am. J. Respir. Crit. Care Med..

[4] McLure L.E.R., Peacock A.J. (2009). Cardiac magnetic resonance imaging for the assessment of the heart and pulmonary circulation in pulmonary hypertension. Eur. Respir. J..

[5] Grünig E., Peacock A.J. (2015). Imaging the heart in pulmonary hypertension: An update. Eur. Respir. Rev..

[6] Yamada Y., Okuda S., Kataoka M., Tanimoto A., Tamura Y., Abe T. (2012). Prognostic value of cardiac magnetic resonance imaging for idiopathic pulmonary arterial hypertension before initiating intravenous prostacyclin therapy. Circ. J..

[7] Swift A.J., Telfer A., Rajaram S., Condliffe R., Marshall H., Capener D. (2014). Dynamic contrast-enhanced magnetic resonance imaging in patients with pulmonary arterial hypertension. Pulm. Circ..

[8] Mathai S.C., Kawut S.M. (2017). Magnetic resonance imaging in pulmonary arterial hypertension. Am. J. Respir. Crit. Care Med..

[9] Galiè N., Humbert M., Vachiery J.-L., Gibbs S., Lang I., Torbicki A. (2015). 2015 ESC/ERS Guidelines for the diagnosis and treatment of pulmonary hypertension. Eur. Respir. J..

[10] La Gerche A., Claessen G., Van De Bruaene A., Pattyn N., Van Cleemput J., Gewillig M. (2013). Cardiac MRI: A new gold standard for ventricular volume quantification during high-intensity exercise. Circ. Cardiovasc. Imaging.

[11] Eduarda M., De Siqueira M., Pozo E., Fernandes V.R., Sengupta P.P., Modesto K. (2016). Characterization and clinical significance of right ventricular mechanics in pulmonary hypertension evaluated with cardiovascular magnetic resonance feature tracking. J. Cardiovasc. Magn. Reson..

[12] Sanz J., Kariisa M., Dellegrottaglie S., Prat-González S., Garcia M.J., Fuster V. (2009). Evaluation of Pulmonary Artery Stiffness in Pulmonary Hypertension With Cardiac Magnetic Resonance. JACC Cardiovasc. Imaging.

[13] Swift A.J., Capener D., Johns C., Hamilton N., Rothman A., Elliot C. (2017). Magnetic resonance imaging in the prognostic evaluation of patients with pulmonary arterial hypertension. Am. J. Respir. Crit. Care Med..

[14] Benza R.L., Miller D.P., Gomberg-Maitland M., Frantz R.P., Foreman A.J., Coffey C.S. (2010). Predicting survival in pulmonary arterial hypertension: Insights from the registry to evaluate early and long-term pulmonary arterial hypertension disease management (REVEAL). Circulation.

[15] D’Alonzo G.E., Barst R.J., Ayres S.M., Bergofsky E.H., Brundage B.H., Detre K.M. (1991). Survival in Patients with Primary Pulmonary Hypertension: Results from a National Prospective Registry. Ann. Intern. Med..

[16] Gall H., Felix J.F., Schneck F.K., Milger K., Sommer N., Voswinckel R. (2017). The Giessen Pulmonary Hypertension Registry: Survival in pulmonary hypertension subgroups. J. Hear Lung Transpl..

[17] Humbert M., Sitbon O., Chaouat A., Bertocchi M., Habib G., Gressin V. (2006). Pulmonary arterial hypertension in France: Results from a national registry. Am. J. Respir. Crit. Care Med..

[18] Humbert M., Sitbon O., Chaouat A., Bertocchi M., Habib G., Gressin V. (2010). Survival in patients with idiopathic, familial, and anorexigen-associated pulmonary arterial hypertension in the modern management era. Circulation.

[19] Wolferen S.A., Marcus J.T., Boonstra A., Marques K.M., Bronzwaer J.G., Spreeuwenberg M.D. (2007). Prognostic value of right ventricular mass, volume, and function in idiopathic pulmonary arterial hypertension. Eur. Hear, J..

[20] Lewis R.A., Johns C.S., Cogliano M., Capener D., Tubman E., Elliot C.A. (2020). Identification of Cardiac MRI Thresholds for Risk Stratification in Pulmonary Arterial Hypertension. Am. J. Respir. Crit. Care Med..

[21] Swift A.J., Rajaram S., Campbell M.J., Hurdman J., Thomas S., Capener D. (2014). Prognostic value of cardiovascular magnetic resonance imaging measurements corrected for age and sex in idiopathic pulmonary arterial hypertension. Circ. Cardiovasc. Imaging.

[22] Schiebler M.L., Bhalla S., Runo J., Jarjour N., Roldan A., Chesler N. (2013). Magnetic resonance and computed tomography imaging of the structural and functional changes of pulmonary arterial hypertension. J. Thorac. Imaging.

[23] Paz R., Mohiaddin R.H., Longmore D.B. (1993). Magnetic resonance assessment of the pulmonary arterial trunk anatomy, flow, pulsatility and distensibility. Eur. Heart J..

[24] Gan C.T.J., Lankhaar J.W., Westerhof N., Marcus J.T., Becker A., Twisk J.W.R. (2007). Noninvasively assessed pulmonary artery stiffness predicts mortality in pulmonary arterial hypertension. Chest.

[25] Padervinskienė L., Krivickienė A., Hoppenot D., Miliauskas S., Basevičius A., Nedzelskienė I. (2019). Prognostic value of left ventricular function and mechanics in pulmonary hypertension: A pilot cardiovascular magnetic resonance feature tracking study. Medicina.

[26] Kallianos K., Brooks G.C., Mukai K., Seguro de Carvalho F., Liu J., Naeger D.M. (2018). Cardiac Magnetic Resonance Evaluation of Left Ventricular Myocardial Strain in Pulmonary Hypertension. Acad. Radiol..

